# Personal determinants of change agents’ decision-making behavior in community health promotion: a qualitative study

**DOI:** 10.1186/s12889-023-16590-y

**Published:** 2023-09-05

**Authors:** Lisa Paulsen, Lea Benz, Christina Müller, Birgit Wallmann-Sperlich, Jens Bucksch

**Affiliations:** 1 Department of Prevention and Health Promotion, Heidelberg University of Education, Keplerstraße 87, 69120 Heidelberg, Germany; 2 Institute of Sport Science, University of Würzburg, Judenbühlweg 11, 97082 Würzburg, Germany

**Keywords:** Determinants, Decision-making, Planning, Implementation, Intervention mapping, Change agents, Community, Local politics, Local government, Health promotion

## Abstract

**Background:**

Implementing environmental changes to promote healthier communities requires initial positive decisions by change agents from local politics and government. However, there is little research on what influences the change agents' decisions. This explorative, qualitative study aims to identify the personal determinants of the decision-making behavior of local change agents.

**Methods:**

We conducted semi-structured interviews to assess the personal determinants of decision-making behavior among 22 change agents from local politics and government. Relevant determinants were identified through a structured content analysis of the interview transcripts using the software MAXQDA 2020.

**Results:**

We found the following seven essential clusters of personal determinants of the decision-making behavior of change agents from local politics and government: Imprinting, socialization, and biography; experiences and involvement; attitudes and outcome expectations towards important issues and aspects; knowledge; emotions; personal benefits; and the perceived influences of others.

**Conclusions:**

The identified personal determinants might serve as a source of understanding the decision-making behavior of change agents in community decision-making processes. Our findings can contribute to the effective planning and implementation of evidence-based multilevel interventions related to changing environmental conditions in communities and provide important information on which personal determinants should be considered when derive strategies for community health promotion within a systematic approach of developing an intervention program theory.

**Supplementary Information:**

The online version contains supplementary material available at 10.1186/s12889-023-16590-y.

## Contributions to the literature

- This study enriches intervention planning models for health promotion by providing a better understanding of environmental change. For this to happen, the personal determinants of change agents must be identified and fundamentally understood to initiate health promotion at the environmental level.
- It is important to learn more about political processes in communities and how to influence relevant health-promoting decisions.
- Our findings provide information that may be transferable to other settings, such as schools and companies, about which personal determinants of change agents need to be changed so that health promotion is set on the agenda.

## Background

Communities are important settings for health promotion. Community health promotion should be based on socio-ecological concepts, which describe that health is not only determined by individual factors, but also by environmental factors that are located at interpersonal, organizational, community, and society levels, or are influenced by an interaction of individual and environmental factors [1–4]. However, environmental-level interventional approaches are often highlighted but seldom used [5–7], and evidence of the intervention effect of changing the physical environment is mixed [8]. From a systematic intervention development perspective, there is a lack of information about how a program theory (or the theory of the problem and the theory of change) in the environmental context can be operationalized. To date, the logical modeling of interventions has more often been explored at the individual level of specific target groups [1, 9, 10].

To create healthy communities, two things are important: 1) Environmental change depends on people and their choices [11]. Therefore, creating healthy communities is usually subject to local decision-making processes and depends on decisions made by actors from local politics and government. 2) The decision-making behavior of these individuals is shaped by a variety of different influencing factors. That’s why, from a program-theoretical perspective it is important to identify the underlying factors influencing the decision-making behavior of these local actors [1, 9]. This is in line with considerations of intervention planning models like Intervention Mapping (IM) [1], the Multilevel Approach to Community Health (MATCH) [2], or the Six Steps in Quality Intervention Development (6SQuID) [10].

Intervention Mapping [1] describes these local actors as so-called environmental or change agents (CAs). Change agents operate at different environmental levels of socio-ecological models and are (mostly) not personally affected by a health problem, but their decision-making behavior can create healthy living conditions and thus influences the health behavior of the target group [1–3]. To be more specific for the change of the physical environment: Decisions of CAs on a physically active-friendly design of a neighborhood, e.g., the construction of a bicycle path, are usually made through democratic processes and can influence the physical activity behavior of the population. Thus, CAs of local politics and government become targets of interventions to which an intervention must be tailored and their decision-making behavior has to be addressed [2]. The group of CAs in local contexts is diverse and not easy to define. They include, among others, the members of municipal or city council as a decision-making body in communities as well as mayors.

In terms of a program theory the determinants of the behavior of CAs have to be identified. Intervention Mapping, for example, requires that at the environmental level the decision-making behavior of CAs needs also to be described by explanatory factors [1]. These explanatory factors are called behavioral determinants [4]. Behavioral determinants can be distinguished into personal determinants, which usually encompass cognitive factors and abilities, such as knowledge, attitude, beliefs, or self-efficacy, and determinants which are found at environmental levels (e.g., social norms, guidelines, and laws) [1]. Kok et al. [12] propose that all behavioral determinants, regardless of their contextual nature, can be fundamentally attributed to the generic accumulation of beliefs. The authors conceptualize beliefs as foundational components within these determinants, which collectively contribute to their overall composition. Consequently, the origins of these determinants can be traced back to individual levels and personal determinants [12]. As we can see, there are some structural conditions according to which CAs have to act (= context factors; e.g., laws or financial resources) and within which CAs behave. The personal determinants (e.g., knowledge), on the other hand, seem to be more directly modifiable or approachable, because they conceal psychological constructs for which there is a large number of methods or techniques for behavior change [1, 2]. Therefore, the focus of this study is on personal determinants of the decision-making behavior of local CAs at the environmental level.

Considering the importance of CAs for environmental change and influencing people’s behavior in general, limited research has been conducted from a public health perspective about what personally determines the decision-making behavior of CAs. While there is a growing body of literature on the importance of physical activity-friendly environments and community health interventions [13–16], less is known about the preceding policy process [17] and, more importantly, the determinants that affect the opinion formation and decision-making of CAs from local politics and government that act on the policy process and lead to the implementation of community health promotion. In fact, the policy-making process is understudied in the field of health promotion [18]; studies have more often focused on determinants for policy implementation [16, 17, 19–25]. Other studies have examined decision-making behavior exclusively, but in different settings, contexts, or with different target groups or different research aims and study designs [26–29].

There is a growing interest in describing the taxonomies of health-promoting interventions, their theoretical approaches, and identified determinants [9, 30]. However, studies mostly focus on the individual behavior change of specific target groups and less on the behavior change of CAs at environmental levels [1, 9, 10, 31]. To effectively use the political arena to implement community health promotion, a basic understanding of how policy-making and especially decision-making work is needed [32]. Therefore, this study is one of the first to explore and identify the personal determinants of the decision-making behavior of CAs from local politics and government from urban and rural contexts in Southern Germany, a rather wealthy region, using a qualitative research design.

## Methods

This qualitative, exploratory study was designed to gain initial insight into the personal determinants influencing the decision-making behavior of CAs from local politics and government from their subjective perspective to create healthy environments. Intervention Mapping recommends qualitative methods, such as interviews, to develop new ideas for determinants or to verify the findings in the research literature [33].

### Sample and recruitment

The sample selection was purposefully guided. Interviewees were recruited via internet research, and based on the identification of local CAs from a preceding stakeholder survey with actors from the field of community health promotion, from two model communities of the research project EUBeKo^1^ [34], funded by the German Federal Ministry of Health. These communities are a city in Baden-Württemberg and a small rural municipality in Bavaria in Germany. Both communities are located in southern Germany, a rather wealthy region. The model city is an industrial metropolis with approximately 300,000 inhabitants, of which almost 50% are women and 15% children and teenagers. Approximately 19% of the population is older than 65 years. Inhabitants with migration background cover almost 48% of the population, mostly from Turkey and Poland. The city has a tight budget situation and a high debt level, which, however, has been quite stable for the past 10 years. The unemployment rate is 6.6%. The city has an above-average proportion of school leavers without qualification with 7.8%, compared to Baden-Württemberg (2019/2020: 4.5%). The municipality in Bavaria has approximately 1,500 inhabitants, with almost 49% women and 17% children and teenagers. 23% of the population is over 65 years old. The proportion of people with a migration background is approximately 1%. The municipality has a relatively low level of debt and the unemployment rate is 1.13%.

We obtained a sample covering a broad range of CAs making decisions at the community level. In Germany, the municipal or city council is the highest decision-making body in communities. But mayors also have some decision-making power within their scope of action. Other bodies, such as the district advisory council, can also influence decisions through their proximity to the citizens and contribute ideas to the municipal/city council or the local government. Although the district advisory council has no formal decision-making authority, it can contribute to the decision-making process in an advisory capacity. County councils can also influence developments in the communities. Although they do not decide which health promotion interventions are implemented in communities, they can influence communities with their expertise. In local governments, heads of offices or departments also have a certain decision-making authority and must be convinced of projects. In this case, employees in the administration can act as "decision preparers" who have to convince their superiors. Other groups who can influence local decisions include citizens, associations, initiatives, experts, interest groups, companies, health insurers, and many more [35]. In this article, we refer exclusively to decision-makers in local politics (municipal/city councils, county councils, and district advisory councils) and local government with leadership responsibilities (mayors, heads of offices, and heads of departments), and not to those preparing decisions or other interest groups. The purposeful selection of interviewees ensured diversity in terms of the political parties and offices responsible for designing environmental change (e.g., sports office, city planning office).

The project staff contacted potential interviewees by telephone or e-mail, through the offices of the various parties or the corresponding secretariats of the administrative offices, or through direct contact with the decision-makers. Interested candidates were informed verbally and in writing about the aims of the qualitative study, the data protection policy, and the interview conditions to obtain their informed consent. We conducted 22 interviews. The study complies with ethical and legal data protection regulations and was approved by the Ethics Committee of the Institute of Sport Science of the University of Würzburg.

### Development of the interview guide

The interview guide was developed between the beginning of April and mid-July 2020 using the SPSS (*sammeln*, *prüfen*, *sortieren*, *subsumieren*, or collecting, checking, sorting, and prioritizing) method of Helfferich [36, 37], and based on the research team's understanding of socio-ecological concepts, behavioral determinants, and decision-making processes in communities, as well as their experience with qualitative research. Besides, similar analyses from previous studies provided ideas for potential questions for the interview guide [38–40]. The interview guide includes a total of 17 open-ended, narrative-generating questions, divided into five thematic groups: I) entry, II) the decision-making process, III) political network analysis/the role of the interviewee and the roles of others in the decision-making process, IV) determinants, and V) conclusion. The complete interview guide can be found in Supplementary file 1. The following interview questions from thematic group “IV) determinants” highlighting the personal determinants of decision-making behavior are relevant to this paper:

- Can you please tell me which factors influence your decisions in general?
- Which personal factors influence your decisions?
- Where exactly does this influencing factor come from?

The interview guide was tested in two pretest interviews with a department head from a municipal department and a politician from a municipal council, on 17/07/2020 and 21/07/2020, respectively. Only minimal adjustments were made to the interview guide after the pretest interviews, so both interviews were included in the main analysis.

### Data collection

An interviewer training session was conducted to ensure that the interviews by different interviewers would be as uniform as possible [41]. Five project staff members interviewed 22 decision-makers from local politics and government between July and December 2020. The interviews were held either face-to-face at the interviewees' workplaces or homes, or by telephone or video conference using Zoom, due to the COVID-19 pandemic. The interviews lasted an average of 60 min, and ranged between 30 and 90 min.

The interviews were transcribed by an external transcription agency. The transcriptions were done verbatim, corrected for dialect and punctuation, and the language was slightly smoothed. The interviews were anonymized, so that no conclusions can be drawn about the personal data of the interviewees.

### Data analysis

From February to October 2021, three project staff members respectively three female junior scientists from the field of public health analyzed the interviews, based on the structured content analysis according to Kuckartz [42], with the help of the software MAXQDA 2020 (VERBI GmbH, Berlin, Germany). In this type of analysis, categories are created, the interviews are analyzed with the help of these categories, the content is structured and summarized, and headings and subtopics are formed [42]. A deductive-inductive approach was adopted to derive the categories. At the beginning of the analysis, the three researchers developed categories based on the interview guide and socio-ecological models (that is, the deductive approach), before reviewing the text material. Subsequently, they derived more categories directly from the empirical material (that is, the inductive approach). Among many other categories of the study, the category of personal determinants emerged from the thematic group of "IV) determinants" of the interview guide. In this article, however, only the personal determinants influencing the decision-making behavior of CAs from local politics and government will be referred to. These deductively and inductively derived categories formed the differentiated category system. The category system contains definitions, quotations, and coding rules to assign the text passages and to distinguish them from other categories [42]. After coding the material, the text passages were paraphrased in parallel by two project members. Discrepancies were discussed and consensus reached. Based on the paraphrasing, a cross-case, thematic analysis was conducted for the personal determinants influencing decision-making behavior and summarized across all interviews [43]. Since an explorative approach was taken, there were no detailed questions in the interview guide about psychological contructs, such as self-efficacy or subjective norm, from which the personal determinants could be derived. Rather, the material was clustered and themes were formed from the project members’ interpretations of patterns of meanings across the interviews, e.g., on the basis of the researchers' prior knowledge of psychological and behavioral determinants. The thematic analysis involved a reflexive reading of the material and the familiarisation with the data formed deeper understanding [44].

## Results

### Sample

The sample consists of 22 decision-makers from local politics and government in the two model communities, seven women and 15 men (Table 1). Of these, 12 come from an urban context and 10 from a rural context. Furthermore, 12 people have a political function and nine have an administrative function, while one person has functions in both areas, as the mayor of a rural community and a member of the county council (Int_9). Another person (Int_10) has a dual political function, and is a member of the municipal council as well as of the county council.

**Table 1:** Overview of the sample (*n* = 22)

ID	Gender	Area	Role	Community
Int_1	Female	Politics	District Advisory Council	Urban
Int_2	Male	Administration	Mayor For Health	Rural
Int_3	Male	Administration	Office Head	Urban
Int_4	Male	Administration	Department Head	Urban
Int_5	Male	Politics	District Advisory Council	Urban
Int_6	Male	Administration	Office Head	Rural
Int_7	Female	Politics	Municipal Council	Rural
Int_8	Female	Politics	Municipal Council	Rural
Int_9	Female	Administration & Politics	Mayor & County Council	Rural
Int_10	Male	Politics	Municipal Council & County Council	Rural
Int_11	Male	Politics	Municipal Council	Rural
Int_12	Male	Administration	Mayor	Rural
Int_13	Male	Politics	Municipal Council	Rural
Int_14	Male	Administration	Mayor	Urban
Int_15	Male	Politics	Municipal Council	Rural
Int_16	Male	Administration	Office Head	Urban
Int_17	Female	Politics	Municipal Council	Urban
Int_18	Male	Politics	Municipal Council	Urban
Int_19	Male	Politics	Municipal Council	Urban
Int_20	Male	Administration	Former Mayor	Urban
Int_21	Female	Administration	Department Head	Urban
Int_22	Female	Politics	Municipal Council	Urban

### Personal determinants of decision-making behavior

**Fig. 1:**
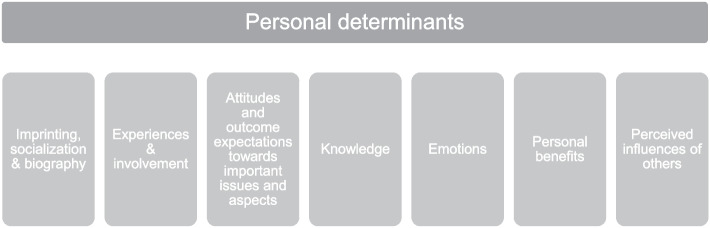
Personal determinants of the decision-making of local change agents

Based on the cross-case thematic analysis, a total of seven clusters of themes or personal determinants were identified as influencing the decision-making behavior of CAs from local politics and government to create healthy environments (Fig. 1). These seven clusters of determinants are: Imprinting, socialization, and biography; experiences and involvement; attitudes and outcome expectations towards important issues and aspects; knowledge; emotions; personal benefits; and the perceived influences of others. These personal determinants are presented in detail below, providing relevant quotes^2^ from the interviews.

### Determinant: imprinting, socialization, and biography

The cluster of determinants “imprinting, socialization, and biography” was derived from the more detailed question about various determinants: "Where exactly does this influencing factor come from?". Since it was specifically asked about, determinants could be identified from almost every interview. This cluster describes how an individual’s personal background (e.g., education and past social environment or professional development and formation of expertise) and the extent to which general personal inclinations or interests (beliefs), faith, morals, and conscience, as well as a person's character affect the formation of opinions and finally the making of decisions:

“The most important factor is definitely my conscience. What I think is right and wrong. So actually I am the biggest factor influencing my decision.” (Int_15, 347–360)

### Determinant: experiences and involvement

The second identified cluster of determinants indicates that experiences and a person's involvement influence the decision-making of CAs. Various aspects were mentioned from almost half of the intervieews here. For example, one's own life experiences, experiences from everyday life (such as living in the district and being familiar with local problems), or one's relationship to the topic (e.g., because of a person's health situation) influence decision-making. In addition, work-related experiences or negative experiences with unnecessary expenses were pointed out:

“The experience that one has had from other projects before, which flows into the decisions.” (Int_2, 863–864)

Also, personal involvement plays a role, for example, involvement in the parents' council, so that the interviewee has the parents' council or kindergarten in mind when making decisions. In addition, being a parent and having kids or older family members influence a person’s decisions:

“Well, I'm on the parents' council, I'm currently still the chairwoman of the parents' council. Yes, of course, I still have my parents' council in mind or the kindergarten in my decisions, of course.” (Int_7, 307–309)

### Determinant: attitudes and outcome expectations towards important issues and aspects

This cluster of determinants includes those issues and aspects that are relevant and valuable to the interviewees and which they would choose over others. Almost all interviewees named determinants in this cluster. The issues and aspects contain general topics, such as topics close to one’s heart, but also specific areas such as education, culture, nature, the environment, sustainability, and animal protection, as well as relevant target groups, such as children and teenagers, and issues that affect a community or organization. Almost half of the respondents mentioned the impact on and welfare of the population as important determinant on decision-making behavior:

“It always depends on the benefit, how many benefit from it and is it useful for the whole community? That's the decision we make. What value does it add to the community? How many people benefit from it?” (Int_9, 338–343)

Change agents are also influenced in their decisions by expected implementation outcomes. Almost all interviewees expect to see certain outcomes after the implementation of projects. These outcomes are weighed up beforehand and influence opinion formation. These expected implementation outcomes can be, for example, feasibility, meaningfulness, plausibility, costs, or demand:

“... you can think about it, even if it costs a lot of money, whether it will be accepted by the population.” (Int_19, 530–531)

### Determinant: knowledge

According to a few interviewees, knowledge is a determinant of decision-making behavior. In their opinion, knowledge includes recent expertise as well as belief in data, numbers, facts, statistics, research, and specialist literature:

“Well, I'm a very numbers-, data-, facts-oriented person. So ultimately, when an employee comes to me and presents something to me and backs it up with numbers, data, and facts, it's relatively easy for me to make a decision.” (Int_16, 538–540)

### Determinant: emotions

The next determinant describes emotions as another influencing factor for the decision-making of CAs. Nearly half of the CAs in local politics and government interviewed make decisions by listening to their gut feeling or following their intuition. They perceive their feelings on a topic or are enthusiastic about it in principle:

“Yes, well, I've already had that experience, sometimes it's also a bit of intuition that you simply perceive some feeling or something. That you then say, okay, there's something special about the case now, and you have to pay a bit more attention to it...” (Int_6, 301–304)

### Determinant: personal benefits

Personal benefits affect the decisions of one CA. Examples from one interview are that a decision-maker’s own business should flourish or that the living environment should be pleasant:

“Well, I didn't start as a district councilor, but as a newly arrived family. […] we opened our business here. Because we live off the people who live in this part of the town. If they are doing well and have money, they can pay us. So, it is our business that the people in the district are doing well and that we don't get a bad reputation here. That also plays a role.” (Int_1, 852–863)

### Determinant: perceived influences of others

The last identified factor that determines decision-making concerns the perceived influences of others (e.g., voters or colleagues), which was named by almost half of the respondents. This determinant can be differentiated into overcoming the influences of others and yielding to the influences of others. Overcoming the influences of others means that the CAs are less influenced by the outside, for example, they vote against something, even if others do not like it, or they are not interested in what their voters would like:

“And I was the only one who voted against it. Not everyone on the council liked it. But I mean, it's going to be built. I know that. Can't do anything about it. Eleven to one. All right. But at least I'm not morally responsible if something goes wrong. If it goes well, it's just the way it is. I've just learned.” (Int_10, 369–373)

Yielding to the influences of others means taking the path of least resistance. The interviewees try to take the perspective of others and how they understand a decision:

“And well, sometimes you really have to say that you also take the path of least resistance. If you have a certain margin of judgment and say you actually have two options, you also ask yourself which decision you can represent better or where will there be less trouble. That is definitely a criterion that is applied every now and then when making a decision.” (Int_6, 280–285)

## Discussion

To our knowledge, this is one of the first studies identifying and understanding personal determinants of the decision-making behavior that influences local decision-making processes to implement community health promotion and to create healthy environments from a public health perspective. The aim was to examine the subjective view of 22 CAs from local politics and government to better understand their decision-making behavior. Overall, it can be concluded that the decision-making behavior of local CAs is determined by a large number of personal determinants. We identified seven clusters of determinants, namely imprinting, socialization, and biography; experiences and involvement; attitudes and outcome expectations towards important issues and aspects; knowledge; emotions; personal benefits; and the perceived influences of others.

Most intervention programs focus only on the individual behavior change of specific target groups and less on the behavior change of CAs to create healthy conditions and environments [1, 9, 10, 31]. However, in the presented study, we were able to find first empirical evidence for the fact that behind decisions on environmental changes there is an organized and intentional human action of CAs and that there is a potential for adressing CAs as target group for interventions [1, 9, 11]. Our findings about the role of personal determinants in explaining decision-making behavior fit with the findings of studies that have also addressed determinants but in different contexts, settings, and with different target groups [25–29]. In addition, our findings confirm the logic of building a program theory in intervention planning using the explanatory power of behavioral determinants. In particular, the use of personal determinants seems to be a promising approach for changes at the environmental level since they seem to be more directly modifiable [1].

Besides this general classification of the results, the seven identified clusters of determinants will be briefly discussed and put into the context of theoretically driven psychological constructs.

In our findings, more important clusters of determinants for opinion formation and decision-making seem to be “imprinting, socialization, and biography” and “attitudes and outcome expectations towards important issues and aspects”, since they were mentioned by almost every respondent. While the cluster “imprinting, socialization, and biography” seems difficult to change, because these aspects are mainly influenced by a person’s past, beliefs, morals, and personality [1], it clearly shows the role of beliefs in determinant formation [12]. In the cluster “attitudes and outcome expectations towards important issues and aspects” the most relevant themes were the impact on and welfare of the population. Health promotion and physical activity were not among their issues of top priority; rather education, nature, the environment, and sustainability were more important. The expected implementation outcomes of interventions are also relevant and were often mentioned. This cluster shows attitudes and outcome expectations towards certain topics (e.g., education or culture), impacts on the population or community (e.g., social balance), and structural aspects or implementation outcomes (e.g., feasibility, costs, and plausibility). Attitudes are positive or negative reactions to something; however, they can include more specific belief constructs, outcome expectations, evaluations of advantages and disadvantages, self-assessments, and motivations for action [1].

Other important clusters of determinants for decision-making appear to be “experiences and involvement”, “emotions”, and "perceived influences of others" with its subthemes of “overcoming the influences of others” and “yielding to the influences of others”, which were adressed by nearly half of the interviewees. The cluster “experiences and involvement” describes, if CAs had a positive experience in a similar situation before (e.g., life or work experience), or if they are personally involved with a certain topic or circumstance (e.g., being a parent, having children). Then, they tend to decide in favor of that topic/situation. The cluster “emotions” can be described as follows: By changing the content of our beliefs, judgments, or ways of thinking, emotions can influence information processing and the final outcome of a decision shows that people categorize and evaluate based on emotions. Moreover, making choices leads to the satisfaction of our needs and the experience of expected emotions [45]. The cluster of determinants "perceived influences of others" with its subthemes of “overcoming the influences of others” and “yielding to the influences of others” allows inferences to be made about known determinants from the literature, such as self-efficacy expectancies. Self-efficacy is often a crucial factor for behavior change and is about whether motivated individuals are able and convinced to change their behavior [1]. The subtheme “overcoming the influences of others” can mean overcoming social influences and subjective norms, which indicates a tendency toward higher self-efficacy. Yielding to the influences of others can provide initial indications of low self-efficacy. However, since these constructs were not specifically queried in this study, we should be cautious when interpreting this finding.

“Knowledge” and “personal benefits” do not appear to be important determinants in making decisions because only a few interviewees raised these topics. Nevertheless, knowledge is a foundation and requirement for most other determinants, such as attitudes, and competencies [1]. But knowledge does not usually lead directly to changes in behavior, nor is it necessarily an easy task to ensure that a target group acquires knowledge [1]. The result that personal benefits do not appear to be mainly relevant in our findings, coincides with the finding above, that almost every respondent mentioned the impact on and welfare of the population as important determinant on decision-making behavior.

Our findings provide an important contribution to the discussion about designing socio-ecological interventions not only as a label but as something that can be systematically intervened in. Our results can be seen as a basis for the explanation of the decision-making behavior of local decision-makers. To change the decision-making behavior of CAs and to put health promotion on the agenda of communities, tailored intervention methods and strategies for behavioral change and persuasion must be derived. As a prerequiste of developing logic models of change and the formation of a program theory of intervention planning models, such as IM, (behavioral) determinants have to be identified [1, 12, 46]. Since CAs are individuals, the determinants of their behavior can be similar to the behavioral determinants at the individual level so that individual intervention techniques (e.g., persuasive communication to change core beliefs) can be integrated [9].

However, these behavior change methods and strategies have yet to be tested for the target group of local CAs in terms of persuasion. Also, determinants are often not, or only barely, identified and described, so it seems impossible to track whether theory-based change methods are the right ones to achieve behavior change [12]. In the sense of evaluation, effect chains, and program theories, it is important to know at which points of the behavior change something worked or did not work to be able to readjust and to see where there were (un)desired side effects that may have led to the behavior change [1, 46]. In this way, resources are not wasted, and tailored solutions can be found [46]. Later, health promoters should be trained in how to convince decision-makers of community health promotion issues and projects. However, this also requires further practice-based evidence and theoretical-conceptual activities [4].

Since this study adopted a qualitative approach, it cannot provide insights into the generalizability of the determinants identified. Rather, it represents a first step in that direction by presenting an empirically derived pool of potentially important determinants whose interrelationships should be investigated in a follow-up study. Similarly, a quantitative assessment is needed to measure the importance of particular determinants and the strength of the correlation between potential determinants and the decision-making behavior of CAs from local politics and government [1]. The results of previous studies that have identified barriers to and facilitators of the implementation of physical activity recommendations or evaluate overarching public health policy decisions could be used as a basis for this purpose [16, 17, 26, 47, 48]. In addition, it would be conceivable to discuss possible determinants of decision-making behavior with experts in a Delphi survey. In this contribution, only personal determinants were identified. Following socio-ecological models, such as in IM, for explaining behavior and behavior change, environmental determinants from interpersonal, organizational, community, and society levels should also be taken into account [1–4, 33]. In addition, it would be interesting to conduct a gendered analysis on the personal determinants of decision-making, as there is much evidence of gendered decision-making processes [49–51].

### Strengths and limitations

The greatest strength of this study lies in the innovativeness of the research question with the identification of personal determinants on the decision-making behavior of local CAs. The study is one of the first to involve CAs from local politics and government and the results highlight the complexity of municipal decision-making processes and the decision-making behavior of CAs. Interview training helped ensure that the different interviewers were able to collect similar data material. Due to the number of interviews (*n* = 22), extensive and detailed data material were collected and analyzed, which ensured the credibility of the study results. Reliability was established by testing whether the category system produced the same results when used repeatedly on the same material. Discrepancies were discussed and consensus was found during the research process. Finally, transferability was ensured by a detailed description of the contextual conditions and the participants interviewed.

However, some limitations of this study need to be acknowledged. First, the CAs’ statements are based on their subjective opinions on determinants influencing their decision-making behavior, which are limited by the size and composition of the sample, as well as their individual contexts and situations. Second, the aspect of social desirability due to the interview situation must also be taken into account, as respondents may not have answered completely honestly.

Third, only the decision-making process described by the respondents was considered. A comparison of their formal correctness based on the corresponding municipal ordinances was dispensed with. Fourth, the majority of the respondents were male. Greater diversity among interviewees and a more balanced ratio of women and men may have led to additional or different personal determinants of decision-making. Fifth, we did not ask about specific psychological constructs, so a vague formulation of the identified themes and personal determinants took place. The interpretation of the determinants was also not easy in some cases and was influenced by the view of the researchers. Sixth, we have translated quotes from German into English. However, the translation does not cause the statements to lose authenticity. Seventh, in the case of the communities that have declared their willingness to cooperate in the EUBeKo project, there could be a positive selection. It is possible that only communities that already had a positive attitude towards the topic of physical activity promotion came forward. We weren’t able to approach other communities. Nevertheless, there were also interview partners who were not so inclined to the topic. Eighth, our study focused on two model communities in Southern Germany, which is – by global standards – wealthy, educated, connected, and safe. That’s why our results cannot be generalized for communities with fractured systems or few resources. Finally, the influence of the researchers on the results should be taken into account. Thus, age, gender, research experience, professional background as well as the choice of methods certainly play a role in the analysis.

## Conclusions

This study provides initial indications of the personal determinants that may influence decision-making and our findings are among the first to focus on CAs from local politics and government in the process of community health promotion. Our approach and further research can contribute to the effective planning and implementation of evidence-based multi-level interventions related to changing environmental conditions in communities. Our findings can provide important information on which determinants should be considered when developing strategies for community health promotion. It is valuable to learn more about the political process in communities and how to influence relevant health-promoting decisions. Our results also indicate information on the decision-making behavior of CAs that may be transferable to other settings, such as schools or companies. While our findings require further research, they present a promising approach to make decision-making behavior a target variable for establishing health promotion as a policy field in the community.

### Supplementary Information

**Additional file 1.** Interview guide: Decision-makers from local politics and government.

## Data Availability

The data described in this article are available from the corresponding author on reasonable request. The complete interview guide can be found in Supplementary file 1.

## Abbreviations

IM: Intervention Mapping
MATCH: Multilevel Approach to Community Health
6SquID: Six Steps in Quality Intervention Development
CA: Change agent

## Footnotes

1 The German acronym EUBeKo stands for Entscheidungs- und Umsetzungsprozesse verhältnisorientierter Bewegungsförderung in der Kommune für mehr Chancengerechtigkeit systematisch planen und implementieren, i.e. Systematically plan and implement decision-making and implementation processes for the promotion of physical activity in the community for more equal opportunities.

2 The quotations were translated from German into English and slightly adjusted for better understanding without losing the meaning of the statement.
