# Supplementary material for: Personal determinants of change agents’ decision-making behavior in community health promotion: a qualitative study
Source: BMC Public Health. 2023 Sep 5;23:1731. doi: 10.1186/s12889-023-16590-y (PMC10481515; doi:10.1186/s12889-023-16590-y)
Supplement: Supplementary file 1 — Additional file 1. Interview guide: Decision-makers from local politics and government. [file 12889_2023_16590_MOESM1_ESM.docx]

**Interview guide: Decision-makers from local politics and government**

| Welcome |
| --- |
| Hello Mr./Mrs. XY, thank you very much for taking your time for the interview. I will record the interview to analyze it later. In the analysis, your data will be anonymized so that your statements cannot be assigned to your person or institution.  Before we begin, I would like to briefly introduce the research project EUBeKo (the German acronym EUBeKo stands for Entscheidungs- und Umsetzungsprozesse verhältnisorientierter Bewegungsförderung in der Kommune für mehr Chancengerechtigkeit systematisch planen und implementieren, i.e. Systematically plan and implement decision-making and implementation processes for the promotion of physical activity in the community for more equal opportunities), in the context of which the interview is being conducted today. The aim of EUBeKo is to identify and analyze the central competencies of actors who promote health and physical activity in communities (e.g., offices of municipal health conferences) and to further develop them. In this context, physical activity is an important aspect of health promotion. For us, promoting physical activity does not only mean that sports courses are available in communities, but above all that the built and natural environment (e.g., barrier-free accesses, playgrounds, bike paths) invites people to be physical active. Such interventions that make a community more physical active-friendly usually involve several actors. We are therefore interested today in how decisions for or against such interventions are made in a community. The results will be transferred into a training concept and a website for municipal practitioners in the sense of competence enhancement. Do you have any questions about the EUBeKo project? |
| Entry  We have two central themes that we are concerned with in this interview, that is the theme of decisions, and the theme of health, or more specifically, physical activity. |
| - To start with, I would be interested to know: What is the importance of health for you personally? - What relevance does physical activity have for you personally? - What do you think influences the physical activity of citizens in your community? - To what extent is physical activity and physical activity promotion currently taken into account in decisions in your community? - To what extent can you personally contribute to a better physical activity behavior among the population in your community? |
| Decision-making process  I have already talked about the term “decision” and would now like to go into this in more depth. In our research project, as already mentioned, we are concerned with the topic of physical activity and physical activity promotion. When we talk about decisions in the further course, those that deal with structural and constructional changes in your community (e.g., green spaces with sports elements, sufficient bicycle paths, or the establishment of networks) are particularly relevant for us in this context. However, this can also mean the preparation of a decision, e.g., from the local government. In addition, we are primarily concerned with your personal and individual attitudes. To answer the following questions, please use a self-selected example, which at best has to do with the promotion of physical activity. |
| - How is a decision made so that physical activity is put on the agenda in your community?   - How is an issue/problem brought to your attention?   - Who brings an issue/problem to your attention?   - What role do your own ideas/issues/problems play and how can you contribute them?   - How do you personally make a decision? Are there any particular strategies or methods? If so, which ones?   - Which people support you in making a decision?   - How is a decision ultimately made in the community?   - How do you know that you personally made the "right" decision?   - What are difficulties in the process of decision-making?   - How do you counter these difficulties? |
| Political network analysis/the role of the interviewee and the roles of others in the decision-making process  Let's now take a look at your role and the role of others in the decision-making process. |
| - To what extent do you feel a personal responsibility to put physical activity promotion on the agenda? - What is your personal role in the decision-making process?   - How would you rate your decision-making power?   - Where do you stand in the decision-making structure in your community? - To what extent can you enforce your decisions? - How can you convince others? - Which other actors, for example from local politics and government, make decisions? - What is your relationship with them? |
| Determinants  Then I would now like to look into the aspect of how exactly you make decisions. |
| - Can you please tell me which factors influence your decisions in general?   - What are other influencing factors?   - What laws or recommendations regarding physical activity and physical activity promotion do you know?   - To what extent do general conditions at federal, state or regional level influence your decisions and can you give examples? (e.g., laws or recommendations)   - What influence do institutional conditions have on your decisions and can you give an example? (e.g., mission statement) - Which personal factors influence your decisions?   🡪 If xy influencing factors are mentioned, ask again: Where exactly does this influencing factor come from? |
| Conclusion |
| - What would I have to do as a citizen to put physical activity on the agenda in your community? - What would I have to do for example, as an employee in the health department or as an office of the municipal health conference, to put physical activity on the agenda in your community? - What would I have to do as a scientist to put physical activity on the agenda in your community? - How do you want to be convinced by me in my respective roles as citizen, actor and scientist? - This brings us to the end of the interview. Is there anything else you would like to tell me?   I would like to thank you very much for your time and for answering my questions. We will inform you about the results and will also send you another online survey in the next few weeks. We would be very pleased if you could also complete it and forward it to your office heads, department heads and municipal council members. |
